# INRIM facilities for HIFU field characterization

**DOI:** 10.1186/2050-5736-2-S1-A5

**Published:** 2014-12-10

**Authors:** Giovanni Durando, Claudio Guglielmone, Chiara Magnetto

**Affiliations:** 1 INRIM, Istituto Nazionale di ricerca metrologica, Torino, Italy

## HIFU power measurement

**Figure 1:**
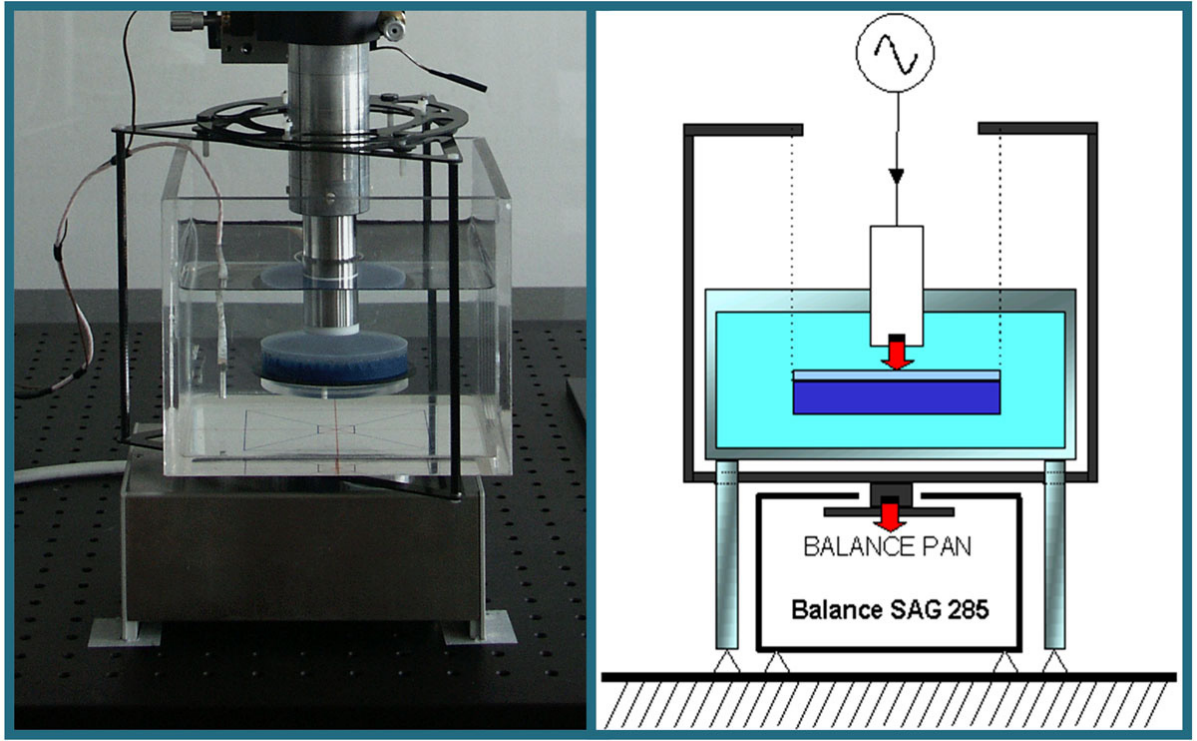
The system based on commercial balance (RFB) realized at INRIM, with a schematic representation.

At ultrasonic laboratory of INRIM (Istituto Nazionale di Ricerca Metrologica) it is possible to measure the ultrasonic power emitted by HIFU (High Intesity Focused Ultrasound) transducers. Two different measuring systems for the determination of ultrasonic power have been developed. Both systems are based on the radiation force balance method. The first system is based on a commercial balance (RFB System) and is used for ultrasonic power from 100 mW to 20 W. The ultrasonic power, *P* , is determined from the measurement of the force exerted on a target by the sound field generated by an ultrasonic source (figure 1).

The second system is based on a submersible load cell (SLC), with full scale sensitivity of 50 g, and it is used for measuring of ultrasonic power from 15 W to 200 W. At high power, heating and permanent damages to the absorbing targets may occur. A load cell with faster dynamic response reduces the insonation time for the measurement and therefore the heating, permits a direct connection with target to the sensing device and the sensor position on the bottom of the vessel simplifies the measurement procedures.

**Figure 2:**
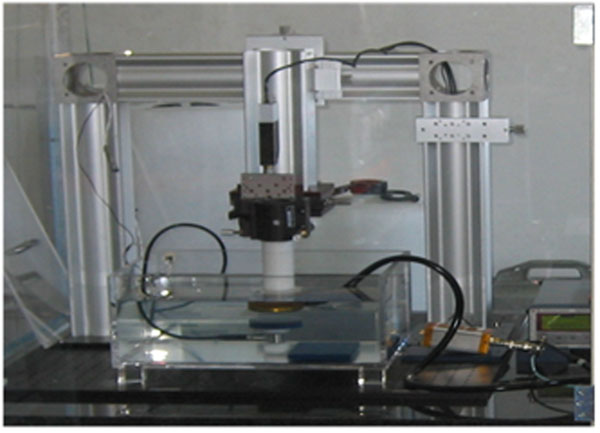
The system based on submersible load cell (SLC) realized at INRIM

## HIFU field characterization

**Figure 3:**
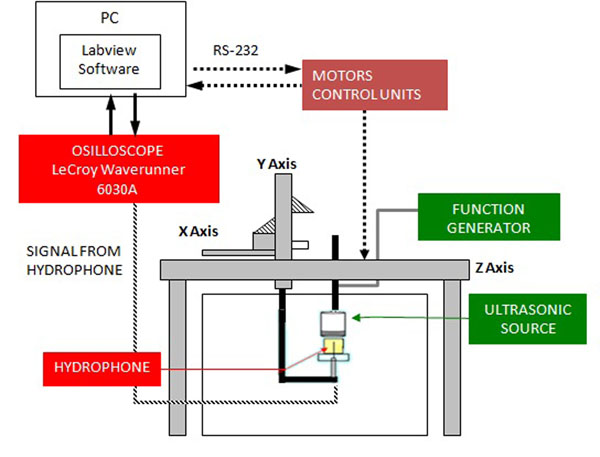
Schematic diagram of the scanning tank system used at INRiM.

The scanning tank system, with a large set of hydrophones (needle, membrane and optical) permits a complete characterization of the HIFU field and the determination of the main safety indexes such as *MI*, *I_spta_*, *I_sppa_*, *TI*. Acoustic characterization of the ultrasound fields is fundamental for the accurate prediction of biological effects induced by ultrasound in tissues and for the development of regulatory standards for clinical HIFU devices. The automated scanning system used at INRIM has been designed to automate the tasks associated with the acquisition, display and computation of data associated with the measurement of ultrasonic field. The system has three motorized axes (x,y,z) each one capable of a few micron resolution, it is supplied with an electronic control module and an automatic software that controls the measuring system (figure 3). Pressure waveforms generated by an HIFU transducer are measured in water: it is however possible to perform measurements also in tissue-mimicking gel phantoms with a fiber optic probe hydrophone. The optic hydrophone allows also the evaluation of the temperature variation induced by ultrasonic field in MRgFUS (Magnetic Resonance guided Focused UltraSound).

